# Inspiratory muscles pre-activation in young swimmers submitted to a tethered swimming test: effects on mechanical, physiological, and skin temperature parameters

**DOI:** 10.1038/s41598-024-52312-z

**Published:** 2024-03-12

**Authors:** Lara Soares de Araujo, Anita Brum Marostegan, Pedro Paulo Menezes Scariot, Juan Bordon Orsi, Carolina Cirino, Marcelo Papoti, Claudio Alexandre Gobatto, Fúlvia Barros Manchado-Gobatto

**Affiliations:** 1 Laboratory of Applied Sport Physiology, School of Applied Sciences, University of Campinas, Pedro Zaccaria Street, 1.300, Jardim Santa Luíza, Limeira, SP 13484-350 Brazil; 2 Study and Research Group in Physiological Sciences and Exercise, School of Physical Education and Sport of Ribeirão Preto, University of São Paulo, Ribeirão Preto, SP Brazil

**Keywords:** Metabolism, Skin models

## Abstract

Inspiratory muscles pre-activation (IM_PA_) has been studied to improve subsequent performance in swimming. However, the effects of IM_PA_ on various parameters in swimmers are still unknown. Therefore, this study aimed to investigate the effects of IM_PA_ on the mechanical parameters, physiological responses, and their possible correlations with swimming performance. A total of 14 young swimmers (aged 16 ± 0 years) underwent a 30-s all-out tethered swimming test, preceded or not by IM_PA,_ a load of 40% of the maximal inspiratory pressure (MIP), and with a volume of 2 sets of 15 repetitions. The mechanical (strength, impulse, and fatigue index) and physiological parameters (skin temperature and lactatemia) and the assessment of perceived exertion and dyspnea were monitored in both protocols. The IM_PA_ used did not increase the swimming force, and skin temperature, decrease blood lactate concentration, or subjective perception of exertion and dyspnea after the high-intensity tethered swimming exercises. Positive correlations were found between mean force and blood lactate (without IM_PA_: r = 0.62, *P* = 0.02; with IM_PA_: r = 0.65, *P* = 0.01). The impulse was positively correlated with blood lactate (without IM_PA_: r = 0.71, *P* < 0.01; with IM_PA_: r = 0.56, *P* = 0.03). Our results suggest that new IM_PA_ protocols, possibly with increased volume, should be developed in order to improve the performance of young swimmers.

## Introduction

The Pre-activation (PA) strategy potentially promotes muscle activation by increasing body temperature to better prepare the body for a subsequent activity, which can improve performance^1,2^. The inspiratory muscles pre-activation (IM_PA_) protocol, which has been the subject of many studies in the field of sports, is a potential way of preceding main efforts and improving physical and sport performance^3–7^. Special attention has been given to this strategy since during high-intensity exercises there is an increase in inspiratory muscles (IMs) activity, leading to hyperventilation, early fatigue and a concomitant decrease in performance^8^, which appears to be minimized when IM_PA_ is used, mainly due to the delay in the inspiratory muscle metaboreflex^9,10^.

In the case of swimming, the IMs are susceptible to adaptations that increase the forced and vital inspiratory volume as a result of the mechanicals movement of the upper limbs for propulsion and immersion of the face in the water, enhancing resistance to fatigue and/or swimming performance^10–12^. Considering that these muscles are very active and to can contribute to the maintenance and quality of swimming^12^, IM_PA_ could be applied in this sport, as it can minimize the effects of double mechanical demand of IMs, improving ventilation and spinal stabilization^13^. However, most studies in this field have focused on IMs training^14–18^ and, to the best of our knowledge, only one investigated IM_PA_^5^, obtaining positive results of this intervention on performance.

To date, the literature lacks positive results on the potential effects of IM_PA_ on physiological parameters in different sports^3,4,7,19–21^, with no studies dedicated to swimming. Taking into account that IM_PA_ can attenuate energy expenditure and heat dissipation through IMs metaboreflex^22,23^, the assessment of blood lactate responses, rating of perceived exertion, and especially skin temperature (ST) could help verify possible changes in physiological parameters.

With the technological advances, it is now possible to obtain thermographic records of athletes' skin in their own training and competition environment is a non-invasive and rapid way^24,25^, assisting in sports to monitoring^26,27^. In addition, blood lactate as a physiological parameter and subjective perceived exertion and dyspnea as psychophysiological measures are widely used after physical exertion as markers to determine the level of exertion and recovery, as they are practical to collect and quick to obtain results^28–31^. Therefore, this gap could be minimized by analyzing these parameters at different times after the application of IM_PA_ with physical effort in swimming.

A protocol that has been widely used to study the physiological and mechanical aspects of high-intensity, short-duration exercises is the 30-s all-out^32,33^. Classically proposed by Bar-or^34^ on a cycle ergometer (Wingate test), this test has been applied to other types of exercise such as running^35,36^ and swimming^37,38^. In the last modality can be applied with the adoption of a sensorized swimming system, which can provide force data and determine impulse and fatigue index accurately, respecting the individuality and specific motor gesture required by the sport^39,40^.

Finally, the 30-s all-out effort is characterized by a predominantly anaerobic stimulus that increases physiological responses such as heart rate and blood lactate production^37,38^. And after this high-intensity test, there is a significant participation of the aerobic metabolism in the recovery process, which ends up restoring energy stores^41^. In this sense, the adoption of the 30-s all-out swimming test seems promising to assess the possible impact of IM_PA_ on the subsequent performance of swimmers, with refined measurements and a high frequency of signal acquisition.

In summary, the present study establishes a connection among knowledge about IM_PA_, physiological measurements and mechanical swimming performance with gaps in the modulation of skin temperature in target regions possibly due to IM_PA_. Through the use of precise tools, we aim to investigate the effects of IM_PA_ on mechanical and physiological parameters, and their possible correlations with tethered swimming performance. Our hypothesis is, that IM_PA_ will enhance swimming performance, consequently increasing force and swimming impulse and reducing the fatigue index during the 30-s all-out test. We also speculate that IM_PA_, combined with swimming warm-up, will increase skin temperature and reduce in the sensation of dyspnea responses that we expect to be positively correlated with performance.

## Methods

### Participants

Fourteen young athletes of both genders (8 males, aged 16 ± 0 years; and 6 females, aged 15 ± 0 years), at competitive levels ranging from regional and state championships in São Paulo, Brazil, to the Brazilian Youth and Junior Swimming Championships (FINA points—SCM: 356 ± 19; LCM: 386 ± 25) participated in the study. Sample size was calculated by G*power software (G*Power version 3.1.9.7 Uiversität Kiel, Germany), that suggested 10 participants to obtain a significant statistical power (sample power 0.95 and α 0.05). As inclusion criteria, we considered athletes with swimming training (at least 2 years) who performed an average of 16 h of training per week, participated in competitions (at least at the regional level), and without history of pulmonary diseases. All procedures were approved by the Research Ethics Committee of the School of Medical Sciences of UNICAMP (protocol no. 39132120.2.0000.5404). The participants and their legal guardians were informed about the procedures and signed the Informed Consent and Assent Form, free and clear. The subjects were instructed not to consume alcoholic beverages or caffeine, not to perform strenuous physical exercises and to maintain a balanced diet throughout the experimental protocol described here in.

### Study design

**Figure 1:**
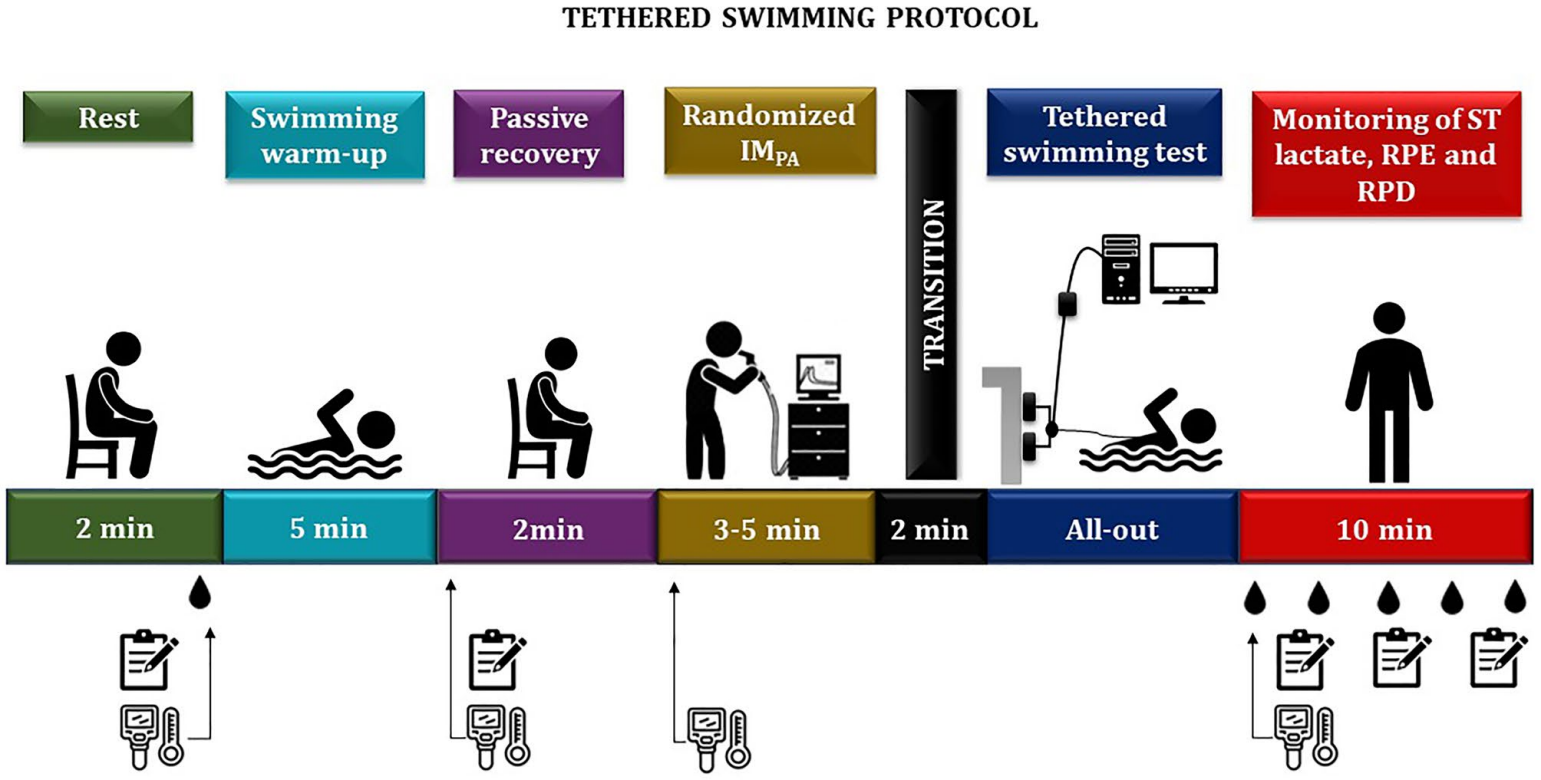
Experimental design used in the study (timeline configuration for the exercise and inspiratory muscle pre-activation (IM_PA_) session). Randomly, each subject underwent two experimental sessions, one in the control condition and the other with IM_PA_. Temporal representation of the interventions performed before, during and after the tethered swimming exercise during which the force recordings occurred. Thermographic records (skin temperature - ST), blood lactate samples and rating of perceived exertion (RPE) and dyspnea (RPD) were obtained during all the assessment sessions in the time windows shown.

All protocols were conducted in the athletes' own training environment. The swimmers were submitted to three assessment sessions, separated by an interval of 24 to 48 h. The first one consisted of the explanation of the research objectives, the application of questionnaires about the participants’ sports history and physical activity level, and the familiarization with equipment (manovacuometer and POWERbreathe® breathing exerciser). Afterwards, they were submitted to anthropometric measurements and assessment of both maximal inspiratory pressure (MIP) and global strength index (S-Index), as detailed below. Lastly, the athletes were familiarized with the ergometer in the pool. In the two subsequent visits, they underwent the all-out tethered swimming test, preceded or not by IM_PA_ at a load equivalent to 40% of the individual MIP (2 sets of 15 repetitions, with 1 a minute interval between sets) (Fig. 1). In both conditions, the swimmers performed a warm-up (WU) with specific swimming gestures (400 m crawl, at intensities from low to moderate), followed by a 5-min passive recovery prior to the tests. The swimming force, impulse, and fatigue index (FI) were collected in these two sessions. The thermographic records were obtained in the last minute of rest, right after the warm-up, before the IM_PA_ protocol, and in the 1st minute of the end of the tethered swimming test. Blood samples were collected at rest, immediately after the all-out effort and at minutes 3, 5, 7 and 10 of passive recovery. Rating of perceived exertion (RPE) and  rating of perceived dyspnea (RPD) were also measured at rest, after WU, after all-out, and at minutes 5 and 10 of passive recovery. All procedures were performed at the same time of day, in a 25 m indoor pool with water temperature maintained at 25 °C, following the rules of the International Swimming Federation (FINA).

### Anthropometric parameters and body composition

For the anthropometric and body composition analysis, the body mass (kg) was measured using a portable digital scale (Multilaser®) with a maximum capacity of 180 kg and precision of (100 g), along with measurements height (cm), body circumferences (cm), wingspan (cm) and humeral and femoral biepicondyle diameters (cm) with the aid of a bone caliper and a Cescorf® flexible anthropometric tape measure. The body fat percentage (%) was estimated following the proposal of Jackson and Pollock^42^ for men and Jackson et al.^43^ for women, where the sum of the seven body folds refers to the sum of the thickness of the pectoral middle axillary skinfolds, triceps, subscapular, abdomen, supra iliac and middle thigh skinfolds for men and, the sum of the thickness of the triceps, thigh, and supra iliac skinfolds (measured by a Cescorf®, adipometer, mm) for women. All measurements were performed by the same evaluator in triplicate, taking the median of the three measurements.

### Maximal inspiratory pressure and S-index determination

The respiratory muscle strength was assessed by MIP (cmH_2_O) using a manovacuometer (± 300 cmH_2_O, GER-AR, São Paulo, SP, Brazil). This non-invasive device has a plastic mouthpiece connected to a small hole (2 mm) to prevent glottic closure. This assessment was conducted by a trained researcher who demonstrated the correct performance of the respiratory maneuver. In the sitting position, the measurement was started from the residual volume and signaled for the athletes to perform a strong and fast inspiration in the total lung capacity. Their nose was occluded throughout the effort. To obtain the best value, all subjects performed three to five attempts, with a 1-min interval between measurements and MIP. The highest value was the one that did not exceed 10% of the difference between attempts^21^. The S-Index (cmH_2_O), was assessed with the aid of an inspiratory device (PowerBreathe K5, IMT Technologies Ltd., UK) in the orthostatic position and with nasal occlusion. Thirty dynamic inspirations were performed with open air flow with the participant exhaling slowly and inhaling the greatest capacity of air quickly and deeply. The peak and mean S-Index corresponded to the highest and average values in the 30 inspirations, respectively^21^. This measurement is derived from mean peak inspiratory flow (PIF) and volume measurements. The MIP and S-Index (mean and peak) measurements are also presented relatized to the athletes' body mass.

### 30-second all-out tethered swimming test

The protocol for determining the mechanical parameters followed the instructions suggested by Papoti et al.^40^, using a tethered swimming system also applied in canoeing^44^. This apparatus consists of a suction cup, a load cell, an elastic cable, a signal amplifier, and software for digital acquisition. The load cell (CSL/ZL250, MK Controle e Instrumentação Ltda™, Brazil), coupled to the suction cup (Vonder™) was attached to the rear exit goal, always maintaining the same goal, by nylon clamps, attached to the elastic cable (Altaflex) laced on the swimmer. In both protocols (with or without IM_PA_), the force signals obtained through load cell deformation were amplified (MKTC5-10®, MK™ control and instrumentation) and processed in a signal conditioning module (USB-6008® National Instruments™). The signals were captured at a frequency of 1000 Hz and transmitted to specific digital acquisition software (LabView Signal Express®, National Instruments™). The system was calibrated before the tests using a dynamometer (Crown®, 20kgf, Oswaldo Filizola Ltda™) in which the load cell was placed in the vertical position and subjected to different force. Regression equations obtained in the calibration procedure were used to convert signals (mv) to force units (N). After the acquisition, the mechanical data, i.e., the variations of force (N), the impulse (N.s), calculated by the area under the curve of the trapezoidal method, and the fatigue index (%), calculated by the equation (maximum force − minimum force)/maximum force × 100, were treated using Microsoft Excel® software.

### Inspiratory muscles pre-activation (IM_PA_) by airflow restriction

After determining the MIP using a manovacuometer and identifying the S-Index, IM_PA_ was performed with the aid of an inspiratory muscle exerciser (POWERbreathe—model K5). The IM_PA_ protocol with airflow restriction was based on the study by Cirino et al.^21^ and Marostegan et al.^36^. The swimmers performed 2 sets of 15 maximum inspirations, with a 1-min interval between the sets and were encouraged to maintain a diaphragmatic inspiratory muscle pattern. The IM_PA_ was performed in the standing position and outside the pool, at 40% of the MIP. Two minutes after the intervention, the athletes were submitted to the tethered swimming test.

### Infrared thermography

Temperature records were obtained by a FLIR® thermographic camera, model One Pro for IOS (19,200 pixels), which has thermal sensitivity to measure temperatures ranging from − 20 to 400 °C, at a resolution of 0.1° W. A specific space was allocated for the thermographic camera to always be positioned in the same place. The recordings were made at an average ambient temperature of 20 °C (2 °C), recorded at the time of collection^24,25,27^. To minimize extrinsic interference, the camera was turned on 30 min before the beginning of the recordings and the participants were kept at rest for a period of 5 min in the recording location, wearing only bathing suits (swimming trunks and sunkini) so that the physiological responses remained stable and the thermal equilibrium was reached. To acquire the images, the athletes were instructed to stand at 2.5–3 m from the thermographic camera (the distance variation depended on the athlete's height). Two images (front and back views) were captured for each moment analyzed in JPG format (640 × 480 pixels) with skin temperature extraction. The skin temperature was quantified by regions of interest, namely, thorax (chest, abdomen and back), upper limbs (biceps brachii and triceps brachii), lower limbs (quadriceps, ischiotibials and triceps surae) and face, using FLIR® Thermal Studio Starter software (version 1.90.10). All records were collected by a single researcher.

### Blood lactate concentration and, rating of perceived exertion and dyspnea

For the analysis of blood lactate concentrations, blood samples (25 µL) were collect from the earlobe, using heparinized and calibrated capillaries. The samples were stored in Eppendorf tubes (1.5 ml) containing 50 μl of 1% sodium fluoride (NaF) and analyzed in an electrochemical Lactate Analyzer (YSI-2300-STAT-Plus™, Yellow Springs, OH, USA Ohio, USA). The Rating of Perceived Exertion (RPE)^30^ and Rating of Perceived Dyspnea (RPD)^45^ were also applied.

### Statistical analysis

All results obtained are expressed as mean ± standard error of the mean (SEM). The normality and homogeneity of the data were tested by Shapiro–Wilk Levene's tests, respectively. Parametric statistics was employed and the normal distribution of data was observed. Two-way ANOVA for repeated measures followed by Newman–Keuls post-hoc was adopted to analyze the effects of pre-activation (with and without IM_PA_) and time on skin temperature in the body regions of interest, blood lactate, RPE, RPD, and strength scores. Student's t-test was used for dependent samples to identify the effect of IM_PA_ on the physiological responses in the post all-out moment and the mechanical outcomes (peak force, mean force, impulse, fatigue index in, absolute values and relativized to body mass). Person's product-moment test was employed to verify the correlation between physiological responses and the results obtained in the tethered test performed with or without IM_PA_. The effect size (ES) and Power (1 − β) were calculated for each paired comparison and two-way ANOVA for repeated measures analyzes from variances. The ES was classified small, medium, and large when ≤ 0.49, 0.5 to 0.79, and ≥ 0.8, respectively. The statistical analyses were performed using STATISTICA® (7.0 version). In all cases, the significance level was set at *P* < 0.05.

### Ethics approval and consent to participate

This study was conducted in agreement with the ethical recommendations of the Declaration of Helsinki and all experiments were approved by the Research Ethics Committee of The School of Medical Sciences (protocol number 39132120.2.0000.5404). After having received information about the experimental procedures and risks, all individual participants (and their legal guardians when under 18 years old) signed an informed consent form declaring to be aware about the study design, the exercise conditions, and the publication of results, and that no details about the individuals would be reported in the manuscript.

## Results

The results of the anthropometric, body composition and inspiratory parameters for male and female swimmers are listed in Table 1, together with the total group results (male and female, n = 14).

**Table 1:** Baseline characteristics of the athletes collected in the 1st assessment session (pre-intervention).

Parameters	Men (n = 8)	Women (n = 6)	Total (n = 14)
Age (years)	16 ± 0	15 ± 0	16 ± 0
Height (cm)	175.3 ± 2.8	161.8 ± 2.1	169.5 ± 2.5
Body mass (kg)	72.1 ± 2.2	66.2 ± 1.9	69.5 ± 1.6
Body fat (%)	15 ± 2	28 ± 1	21 ± 2
Respiratory muscle strength			
MIP (cmH_2_O)	140.0 ± 12.0	102.5 ± 4.4	123.9 ± 8.6
Relative MIP (cmH_2_O kg^−1^)	1.9 ± 0.1	1.6 ± 0.1	1.8 ± 0.1
Mean S-Index (cmH_2_O)	127.8 ± 7.4	77.7 ± 4.4	101.1 ± 8.2
Relative mean S-Index (cmH_2_O kg^−1^)	1.8 ± 0.1	1.2 ± 0.1	1.5 ± 0.1
Peak S-Index (cmH_2_O)	143.4 ± 6.9	94.9 ± 3.2	117.0 ± 7.9
Relative peak S-Index (cmH_2_O kg^−1^)	2.0 ± 0.1	1.4 ± 0.1	1.7 ± 0.1

MIP: maximal inspiratory pressure; S-Index: global strength index; Results (n = 14) expressed as mean ± SEM.

### Mechanical parameters

**Figure 2:**
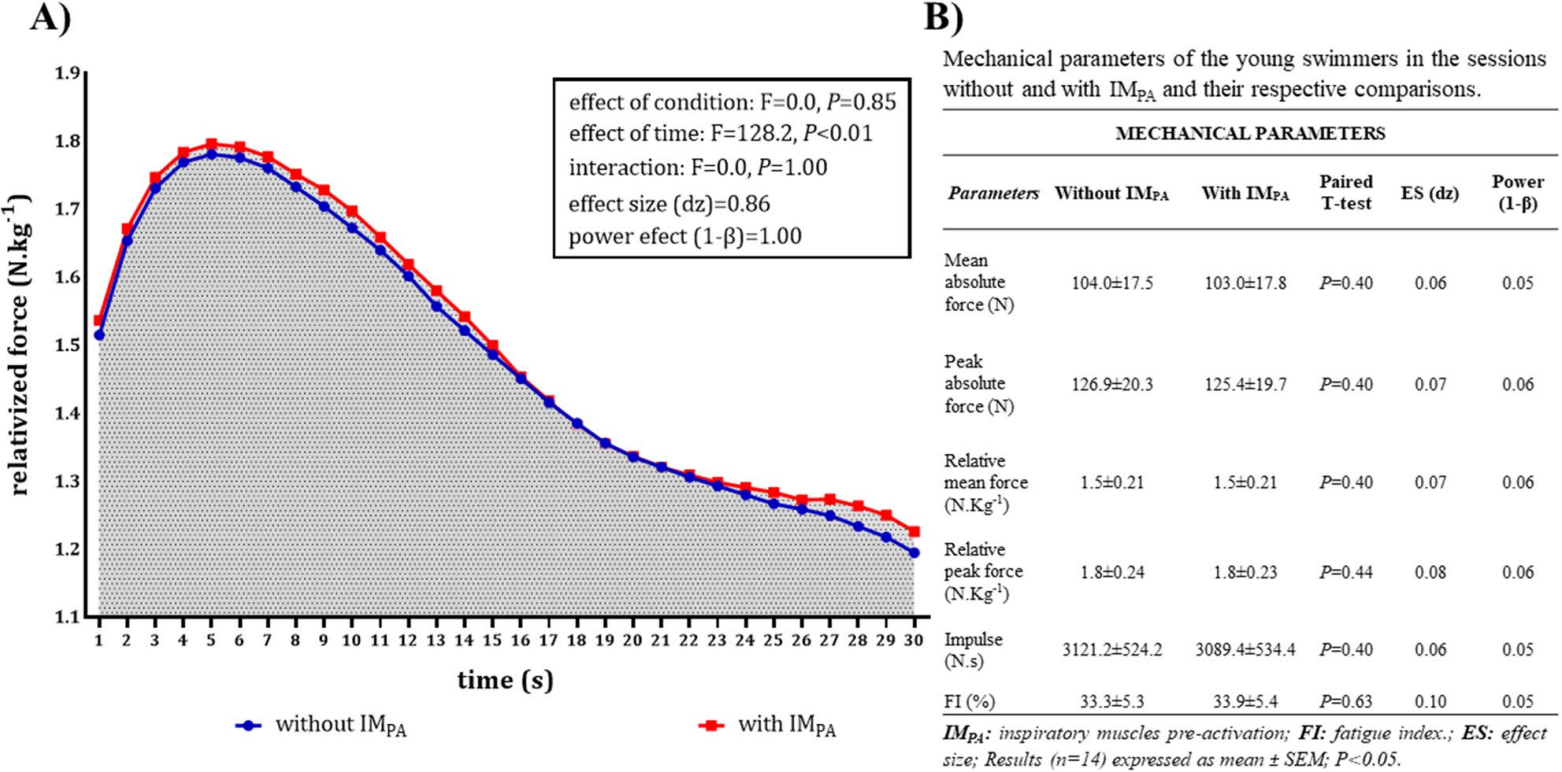
(**A**) Force relativized to body mass (N kg^−1^) during the tethered swimming trials (all-out) with and without IM_PA_. The impulse (N s) is represented in grey by the area over the curve of the trapezoidal method. (**B**) Table of the mechanical parameters with and without IM_PA_. Values taken as mean and statistical difference in interaction with *P* < 0.05 (n = 14).

The results of relativized force (N.kg^−1^) for the total number of participants (n = 14) are illustrated in Fig. 2A, which shows the relativized mean force in each second during the all-out tethered swimming test (30 s) for both sessions (without and with IM_PA_). No significant differences were found between the relativized force values of the sessions with and without IM_PA_ during the all-out tethered swimming test.

Figure 2B compares the mechanical data results (i.e., mean absolute force, peak absolute force, relative mean force, relative peak force, impulse, and fatigue index) between the sessions performed without and with IM_PA_. No difference was observed between the sessions for any of the mechanical variables studied.

### Physiological parameters

Table 2 presents the physiological and perceptual results measured immediately after the exercise sessions performed without and with IM_PA_. For all variables reported, total skin temperature, blood lactate, and rating of perceived exertion and dyspnea showed no significant differences between the sessions.

**Table 2:** Physiological parameters immediately after the all-out effort in two experimental sessions (without and with IM_PA_) and their respective comparisons.

Physiological parameters after all-out					
Parameters	Without IM_PA_	With IM_PA_	Paired T-test	ES (dz)	Power (1 − β)
Total ST (C°)	32.8 ± 1.2	33.0 ± 1.3	*P* = 0.38	0.17	0.09
Blood lactate (mM)	8.72 ± 1.84	8.40 ± 1.80	*P*= 0.54	0.18	0.10
RPE (0–10)	9 ± 1	8 ± 1	*P*= 0.58	0.10	0.06
RPD (0–10)	8 ± 1	8 ± 2	*P*= 0.53	0.13	0.07

IM_PA_: inspiratory muscles pre-activation; ST: skin temperature; RPE: rating of perceived exertion RPD: rating of perceived dyspnea; ES: effect size. Results (n = 14) expressed as mean ± SEM; *P*< 0.05.

**Figure 3:**
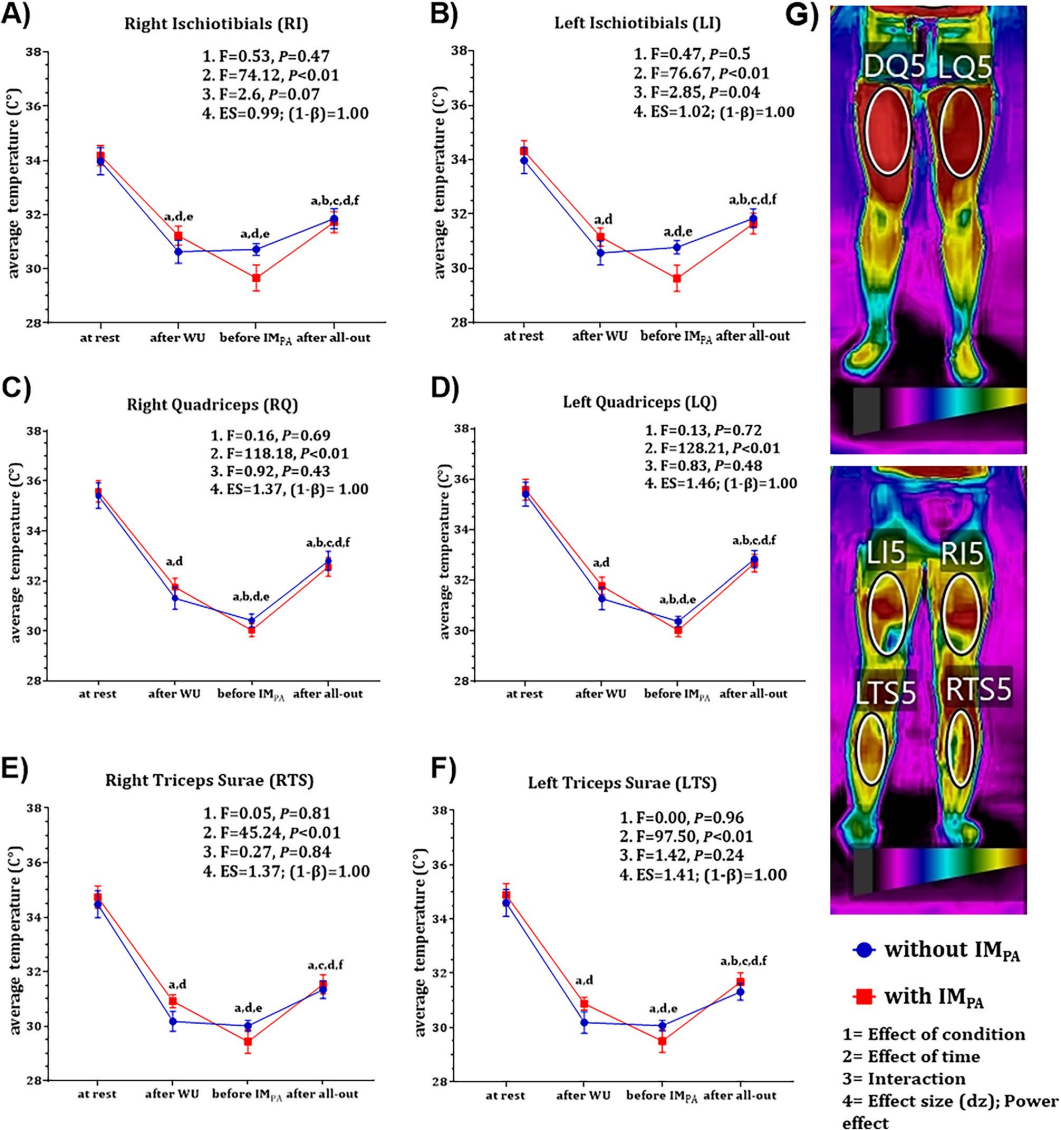
Lower limb skin temperature (mean ± standard error of the mean) at four collected time points (at rest, after warm-up, before IM_PA_ and after the all-out swimming test) in different conditions (with and without IM_PA_). **A**, **D** = significant difference compared to rest; b, e = significant difference compared to the time after WU; and **C**, **F** = significant difference compared to the time before IM_PA_ application; (**G**) anterior and posterior views of the lower limb skin temperature in the anatomical position; *P* < 0.05.

**Figure 4:**
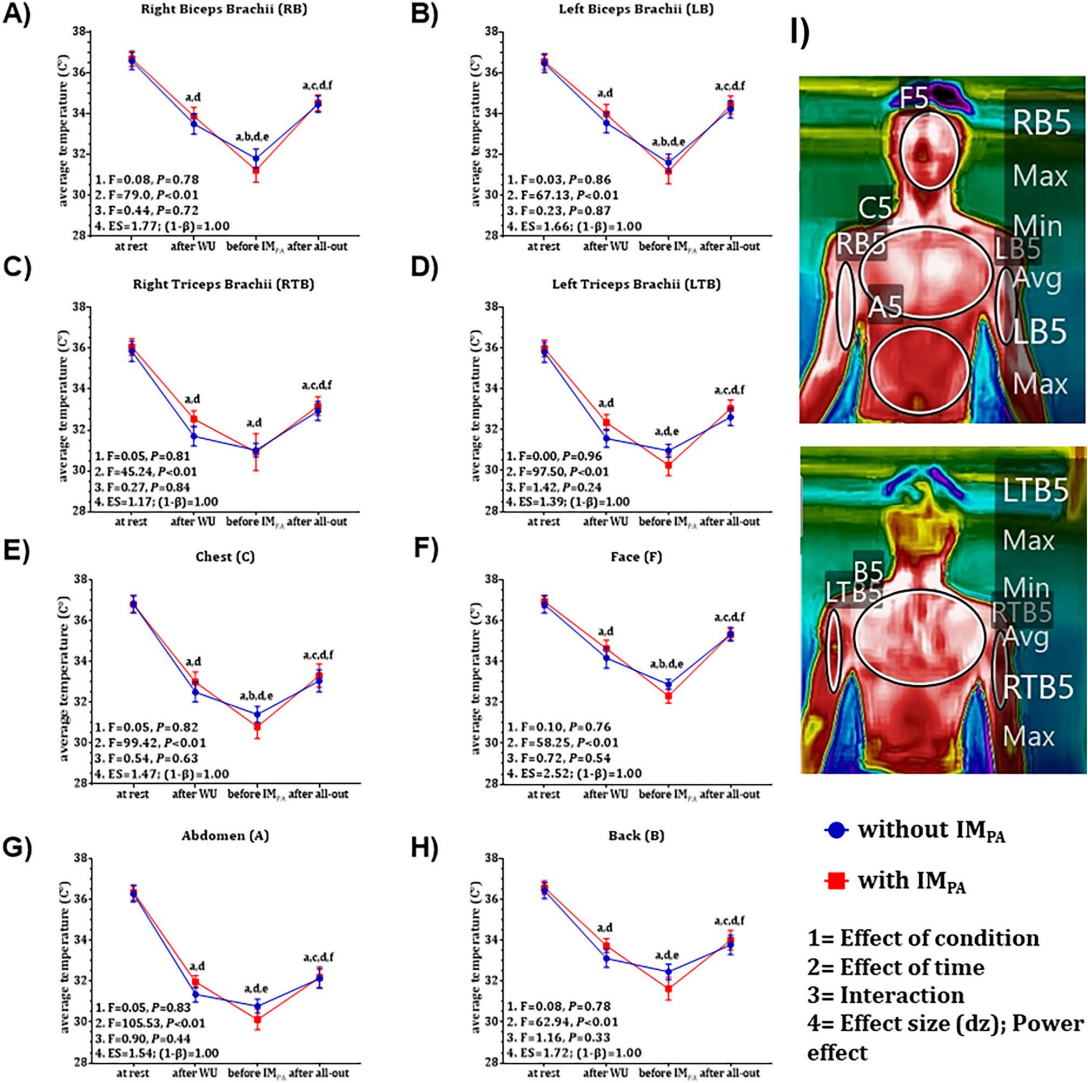
Upper limb skin temperature expressed as mean ± standard error of the mean at four collected times (at rest, after warm-up, before IM_PA_ and after the all-out swimming test in different conditions (with and without IM_PA_). **A**, **D** = significant difference compared to rest; b, e = significant difference compared to the moment after WU; and **C**, **F** = significant difference compared to the moment before IM_PA_ application; (**I**) anterior and posterior views of the upper limb skin temperature in the anatomical position; *P* < 0.05.

The skin temperature results observed in the lower and upper limbs are shown in Figs. 3 and 4, respectively. There was no effect of IM_PA_ on total skin temperature (F = 0.24; *P* > 0.05) and no interaction between the skin temperature values at different times for both conditions (with and without IM_PA_), except for the left ischiotibial (LI) temperature (F = 2.85; *P*= 0.04), which indicates that the thermal variation over time was different between the sessions. However, the post hoc result reveals, that the difference was evidenced immediately before IM_PA_ application in the post all-out moment in both conditions, viewed separately (without IM_PA_: *P* < 0.05; with IM_PA_: *P*< 0.01). The effect of time on skin temperature was observed in different lower and upper muscle limbs (Figs. 3 and 4, respectively).

**Figure 5:**
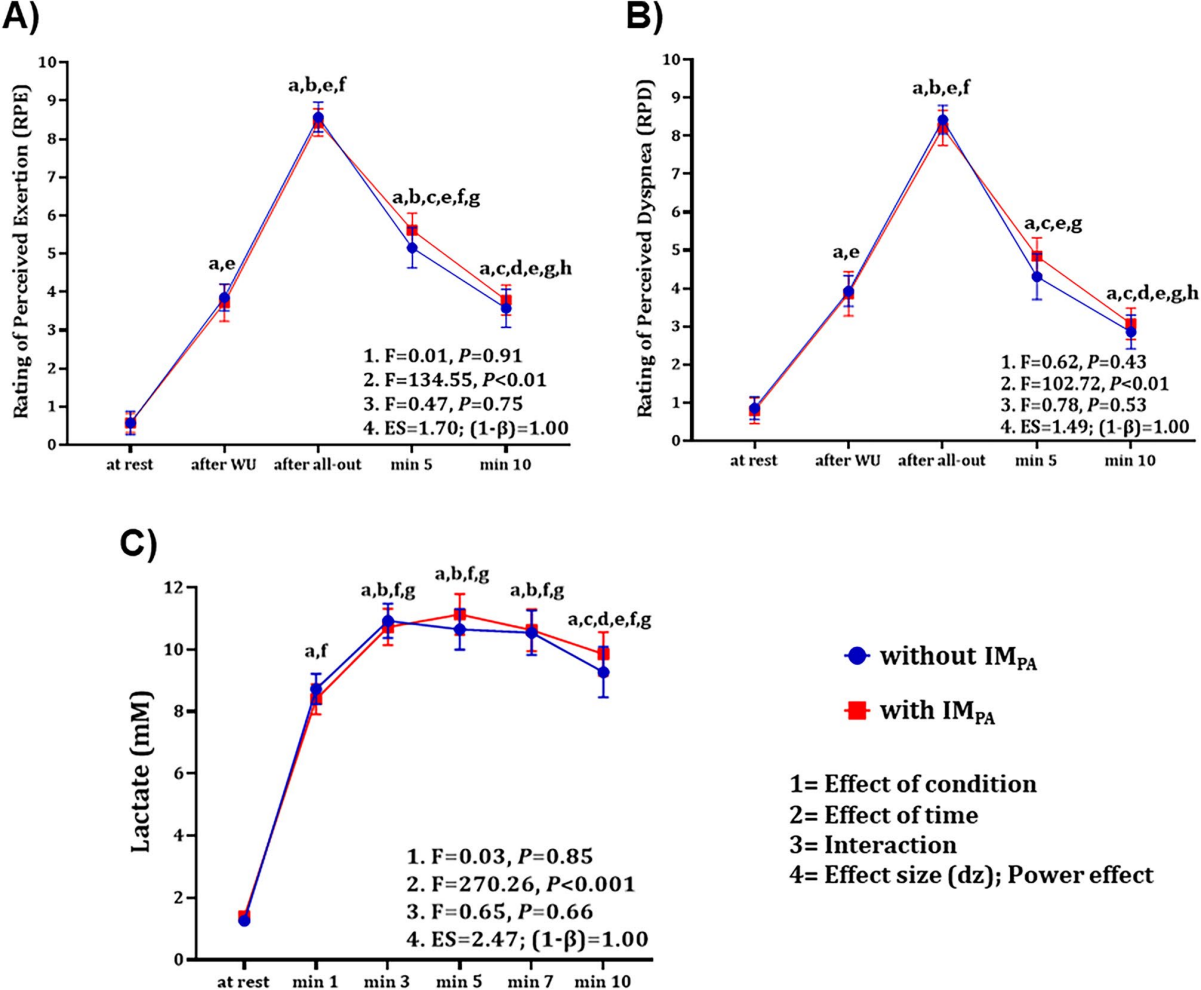
Physiological and psychophysiological parameters before and after the all-out tethered swimming test. (**A**) Rating of perceived exertion and (**B**) rating perceived dyspnea without and with IM_PA_: a, e = significant difference in relation to rest; b, f = significant difference in relation to the moment after WU; c, g = significant difference in relation to the moment after the all-out swimming test; and d, h = significant difference in relation to minute 5. (**C**) Blood lactate (mM) without and with IM_PA_: a, f = significant difference with respect to rest; b, g = significant difference with respect to minute 1; c = significant difference with respect to minute 3; d = significant difference with respect to minute 5; and e = significant difference with respect to minute 7. Results expressed as mean and standard error of the mean; *P* < 0.05.

Finally, the results of the rating of perceived exertion, rating of perceived dyspnea and lactatemia are displayed in Fig. 5. There were no statistical differences between the conditions (without and with IM_PA_) for these responses and no interaction between this effect and time. The exception is the effect of time found when comparing moments within the same session.

### Correlations between mechanical and physiological parameters

Person's product-moment test was used to identify possible significant correlations between mechanical and physiological responses (Table 3). The results indicated significant correlations between mean force and blood lactate responses for all sessions and RPE for the session without IM_PA_, in addition to significant correlations between impulse and lactate for all sessions. Nonetheless, no significant correlation was found between fatigue index and the other physiological parameters assessed at the end of the tethered swimming test.

**Table 3:** Correlations between mechanical and physiological parameters obtained immediately after the all-out swimming test in both conditions (without and with IM_PA_).

Physiological variable	Without IM_PA_^14^	With IM_PA_^14^
Mean force		
Total ST (C°)	r = 0.46, *P* = 0.09	r = 0.40, *P* = 0.15
Blood lactate (mM)	r = 0.62, *P* = 0.02	r = 0.65, *P* = 0.01
RPE (0–10)	r = − 0.58, *P* = 0.03	r = − 0.42, *P* = 0.14
RPD (0–10)	r = − 0.21, *P* = 0.47	r = 0.03, *P* = 0.91
Impulse		
Total ST (C°)	r = 0.34, *P* = 0.22	r = 0.29, *P* = 0.30
Blood lactate (mM)	r = 0.71, *P* < 0.01	r = 0.56, *P* = 0.03
RPE (0–10)	r = − 0.29, *P* = 0.32	r = − 0.32, *P* = 0.26
RPD (0–10)	r = 0.01, *P* = 0.96	r = 0.04, *P* = 0.90
Fatigue index		
Total ST (C°)	r = 0.15, *P* = 0.61	r = 0.12, *P* = 0.68
Blood lactate (mM)	r = 0.09, *P* = 0.75	r = 0.12, *P* = 0.67
RPE (0–10)	r = − 0.32, *P* = 0.26	r = − 0.36, *P* = 0.21
RPD (0–10)	r = 0.01, *P* = 0.97	r = − 0.15, *P* = 0.61

ST: skin temperature; RPE: rating of perceived exertion; RPD: rating of perceived dyspnea; *P < 0.05.

## Discussion

Our study analyzed the effects of IM_PA_ on the physiological (ST, lactacidemia, RPE and RPD) and mechanical (strength, impulse, and FI) parameters in an all-out swimming test. Contrasting with our hypothesis, IM_PA_ did not improve the performance of young swimmers subjected to a tethered swimming test, since no significant differences were observed, both in the mechanical parameters during the test and in the physiological responses after the protocol (ST in different regions of the body, total ST, lactacidemia, RPE and RPD). Significant correlations between force and blood lactate were identified. There was also a significant correlation between impulse and blood lactate concentrations in both tests (preceded or not by IM_PA_).

Swimming predominantly involves the activation of the upper body muscles, thus requiring greater muscle force to maintain the swimming quality^46^. Based on this idea, with the application of IM_PA_ the demand for swimming force would be attenuated by the increase in blood flow to the locomotor muscles during the activity, which was not demonstrated herein. Our results differ from those reported in studies conducted with the same PA protocol in running^36^ and handball^10^. What distinguishes this sport from those performed on the ground is the position of the body, in which the subject remains horizontal throughout the activity. In addition, hydrostatic pressure acts on the chest wall, resulting in higher inspiratory pressures without requiring the individual to increase the work of the diaphragm^12^.

Considering the above, our hypothesis may not have been confirmed due to the increase in pleural pressure and lung volume, as the individual may simply be floating in the water, causing changes in their physical responses in order to adapt to an environment that is not natural for the body^12^. It should also be noted that, although important for the refined acquisition of mechanical signals, in this study the athletes were tethered to an elastic cable, which also increases the load imposed during swimming. This differs from the work developed by Wilson et al.^5^, who reported positive effects of IM_PA_ on the swimmers' performance, but in freestyle swimming. In our case, it is possible that swimming in a tethered position may have contributed, albeit involuntarily, to an elevated inspiratory pressure during the stroke—an effect that was not overridden with the application of the IM_PA_ protocol.

Furthermore, in the case of tethered swimming, the physical demands of the test are associated with the predominant use of alactic and lactic anaerobic capacities since the effort is maximal, characterized by high intensity and short duration (30 s all-out)^47^. During high-intensity efforts, the body increases the cardiac output, which in turn elevates the pulmonary vascular pressure and restricts O_2_ transport, increasing IM fatigue. The inspiratory metaboreflex ultimately impairs the redistribution of blood to the most active muscles during exercise^48,49^. According to the literature, the attenuation of the IM metaboreflex with IM_PA_ would improve performance by reducing energy expenditure and heat dissipation^22,23^, which was not observed in our results.

In addition to performance-related variables, studies conducted in sports other than swimming reported positive effects on physiological responses when different IM_PA_ protocols were used^3,4,6,20,36^. In these studies, the only parameters evaluated that were similar to those used herein were RPE and RPD, and in our specific case, there were no differences between the two experimental conditions (without and with IM_PA_). The RPE examined showed an inverse correlation with mean force for the session without IM_PA_ since the participants who showed lower force scores reported higher RPE values. Moreover, the greater the perceived intensity of breathlessness, the greater the maximum force used by the IMs to breathe in proportional values^50^, resulting in higher blood flow to deliver O_2_ and maintain or increase IM strength, and consequently compromising blood flow to the locomotor muscles^51^. Lastly, there was no correlation between swimming force and RPE and RPD in the session with IM_PA_, suggesting that the IM_PA_ proposed herein slowed, perhaps in a simple way, the increase in blood flow to the IMs because these muscles were already activated.

In our study, even though lactacidemia was not influenced by the application of IM_PA_, a positive and significant correlation was found between blood lactate and force and impulse, confirming that the individuals who performed better in the tethered test stimulated the lactic anaerobic pathway in a more pronounced way. These results are consistent with those found in the literature, evidencing that post-exercise blood lactate is a critical marker of the level of exertion and recovery and that this response is directly related to the physical effort exerted^28,29^. Other studies also failed to show positive effects of IM_PA_ on the physiological variables analyzed^3,4,7,19–21^. On the other hand, from the qualitative results it was possible to observe positive responses when the athletes were asked about their performance in the tests. Several reported "greater ease of swimming", "longer apnea time during swimming", "faster recovery from fatigue at rest" and "less breathlessness" when the efforts were performed in the IM_PA_ session. These reports are similar to those found in other studies using respiratory equipment for IM training, in which the participants reported "less feeling of dyspnea" and "less chest tightness during exercise"^20,52^.

Regarding the results obtained from skin temperature measurements analyzed in a segmented manner in specific regions, we again did not observe the effect of IM_PA_, contrary to our expectations. Greater heat production is strongly related to increased activity of the muscles during effort and exercise intensity^25,53,54^. Additionally, a proportional increase in muscle temperature can be associated with an enhancement in performance, favoring muscle metabolism and increasing the speed of nerve impulse transmission to the muscles, strength, and agility^1,55^. On the other hand, during physical activity the skin temperature does not respond in the same way as the muscle temperature^54,55^. During swimming, there is a decrease in temperature compared to the responses before the beginning of swimming due to the contact of the body with water and an abrupt increase in temperature when the swimmer leaves the water^56^. This behavior was observed in our study in the different skin regions analyzed (Figs. 4 and 5), but without any changes caused by the respiratory intervention before the swimming effort.

One factor that may limit the assessment of temperature in the swimming modality is the clothing worn by the athletes. In the present study, although the athletes used towels to dry themselves after the test so that the thermographic images could be taken, the swimwear and/or sunkini evaluated remained damp, with the residual water running down the body. Despite this limitation and the non-identification of the effects of IM_PA_ on skin temperature responses, we suggest the inclusion of this measure in future investigations involving swimming so as to allow these findings to be further explored in a sporting context^53^.

Another limiting factor of our study is that the intervention chosen (2 × 15 at 40% of the individual MIP with a 1-min rest interval between sets) did not positively modify the dependent variables studied for the target sport. The choice of protocol was based on the positive PA findings in other sports^21,36,57^, as well as its easy applicability and short execution time. The vast majority of studies in the current literature use a volume of 2 sets of 30 repetitions, with positive effects on physiological, inspiratory, and mechanical parameters^9,12^. The load used in our protocol (40% of the MIP) was in line with previous studies that selected this inspiratory load because it represents the highest intensity at which inspiratory muscle fatigue is not induced^9,58^.

In contrast, Wilson et al.^5^ applied IM_PA_ in conjunction with a swimming-specific warm-up (without an interval between the overall warm-up and IM_PA_) with a volume of 2 sets of 30 repetitions to evaluate professional swimmers who performed IM_PA_ and then a 100 m freestyle test compared to a control group. On this occasion, the authors found a reduction in the 100 m test time when the swimmers performed the effort post to the IM_PA_ protocol. Considering that the IMs of swimmers are already highly demanded during swimming^12^, resulting in increased IM strength and lung size compared to land-based athletes^59^, the shorter IM_PA_ strategy (2 × 15 repetitions) may not have been characterized as a sufficient stimulus to induce a brief but effective effort of the IM to improve swimming performance based on an anaerobic stimulus.

Like other investigations^7,60^, our study did not find positive effects of IM_PA_ on performance and physiological responses. Nevertheless, our study is the pioneer in the application of IM_PA_ in tethered swimming, in an attempt to associate mechanical and physiological variables in order to expand knowledge of the effects of this warm-up strategy. We also made progress in skin temperature monitoring, which requires specific equipment for detection. In conclusion, IM_PA_ can be incorporated into training routines and competitions since it is a mobile, easy to use, non-invasive, legal, and safe protocol. Therefore, we emphasize the importance of further studies using IM_PA_ in swimming, so as to expand the application of this stimulus to different categories, including before other longer swimming trials, both in tethered and freestyle, thus contributing to the scientific advancement of this modality and improving the performance of swimmers.

## Conclusions

Our results indicate that the inspiratory warm-up protocol (2 sets of 15 repetitions with a 1 min interval) at 40% of the MIP did not improve the force of swimmers in a high-intensity (30 s all-out) tethered swimming protocol. In addition, IM_PA_, at least as applied here, did not increase skin temperature, reduce blood lactate concentrations, or rating of perceived exertion and dyspnea immediately after the tethered swimming test. On the other hand, positive reports of the use of inspiratory muscles pre-activation were signaled by the athletes, reinforcing the importance of further studies using this strategy in swimming.

## Data Availability

All relevant data are in the manuscript. For additional information, the data will be made available upon request by contacting the corresponding author (Fulvia B Manchado-Gobatto).
